# Central adiposity and sex influence lipid and glucose indices in Mexican adults: implications for metabolic risk assessment

**DOI:** 10.3389/fnut.2026.1834724

**Published:** 2026-08-26

**Authors:** César Hernández-Guerrero, José P. Tejeda-Miramontes, Edgar J. Mendivil, Jaime García-Mena, Erika Arenas, Hugo Ramírez-Saad, Graciela Teruel

**Affiliations:** 1 Departamento de Salud, Universidad Iberoamericana Ciudad de México, Mexico City, Mexico; 2 Departamento de Genética y Biología Molecular, Centro de Investigación y de Estudios Avanzados del Instituto Politécnico Nacional, Mexico City, Mexico; 3 Department of Sociology, University of California, Santa Barbara, Santa Barbara, CA, United States; 4 Departamento de Sistemas Biológicos, Universidad Autónoma Metropolitana-Xochimilco, Mexico City, Mexico; 5 División de Estudios Sociales, Universidad Iberoamericana Ciudad de México, Mexico City, Mexico

**Keywords:** atherogenic index of plasma, central adiposity, genetic risk score, metabolic heterogeneity, sex differences, triglyceride–glucose index, waist-to-height ratio

## Abstract

**Introduction:**

Central adiposity is a major determinant of cardiometabolic risk. However, routinely used composite lipid–glucose indices are often interpreted to indicate a uniform metabolic signal across individuals with different body fat distributions. This study examined whether the interpretive behavior of the atherogenic index of plasma (AIP) and the triglyceride–glucose (TyG) index varies with central adiposity and the evaluated three-variant triglyceride-weighted genetic risk score (wGRS) in Mexican adults.

**Methods:**

A cross-sectional analytical design was applied to data from 396 participants stratified by waist-to-height ratio (WHtR), incorporating correlation structure, an exploratory WHtR × wGRS interaction, and distributional analyses.

**Results:**

Across WHtR tertiles, AIP was higher in the upper tertiles than in the lowest, with minimal additional separation between the intermediate and highest groups, and TyG showed the same upper-versus-lower tertile pattern. Sex-stratified predictions from the continuous interaction models showed that the absolute difference in AIP between wGRS levels of −1 SD and +1 SD narrowed from 0.91 to 0.15 z-score units in men and from 0.32 to 0.03 in women as WHtR increased from −1 SD to +1 SD. Distributional analyses revealed persistent within-stratum overlap, indicating metabolic heterogeneity despite similar anthropometric classifications.

**Discussion:**

Identical AIP or TyG values may occur across different adiposity levels and degrees of wGRS-related separation within the evaluated population, supporting interpretation of these indices in relation to central adiposity.

## 1 Introduction

Cardiometabolic indices, such as the atherogenic index of plasma (AIP) and triglyceride–glucose (TyG) index, are widely used in nutritional and epidemiological research to summarize lipid–glucose interactions and characterize metabolic risk (1). These markers are frequently interpreted as reflecting a comparable biological signal across individuals, despite differences in fat distribution. However, central adiposity influences lipid turnover, insulin dynamics, and inflammatory activity, suggesting that identical index values correspond to different metabolic contexts (2, 3). Most prior investigations have emphasized predictive discrimination through ROC analyses and cut-off values, rather than evaluating how these indices behave within comparable adiposity strata (4). Accordingly, their stability across central adiposity gradients has not yet been formally evaluated.

Composite indices derived from triglycerides and glucose, including continuous TyG-related formulations, have been combined or compared to enhance statistical discrimination (5). However, AIP and TyG share triglyceride information, raising the possibility that apparent improvements reflect structural coupling, rather than independent biological domains. High correlations among triglyceride-derived metrics are seldom evaluated beyond average associations or area under the curve (AUC) comparisons (4, 5). Such emphasis on discrimination metrics does not determine whether the indices capture distinct metabolic dimensions. Consequently, it remains unclear whether these markers provide complementary contextual insights or primarily capture overlapping triglyceride-driven variations. Clarifying their joint structures may refine the interpretation of routinely available composite markers across adiposity levels.

Genetic studies have evaluated interactions between adiposity and polygenic scores using mean triglyceride or LDL-related traits (6, 7). Studies in Mexican and European cohorts reported variation in lipid-related genetic associations across anthropometric levels (6, 8). These analyses focus on mean trait differences rather than on whether genetic separation remains discernible when central adiposity is high and lipid traits are summarized through composite indices. If central fat accumulation reduces phenotypic dispersion, the separation between genetic strata may also diminish when evaluated using integrated markers. Whether this contrast persists in the presence of high central adiposity remains unclear.

Obesity is increasingly recognized as heterogeneous, with depot-specific, genetic, and clustering-based subtypes exhibiting divergent cardiometabolic profiles (9–11). Metabolic signature analyses have demonstrated that adiposity indices correspond to overlapping yet distinct biochemical characteristics, often varying by sex (12). Epidemiological frameworks indicate that relevant heterogeneity may reside in distributional tails rather than exclusively in the central tendency (13, 14). These observations suggest that similar anthropometric classifications encompass diverse metabolic profiles. These considerations are particularly pertinent in Mexican populations, which are characterized by a high prevalence of central adiposity, an elevated triglyceride burden, and an admixed genetic architecture (2, 8). Evaluating distributional behavior within anthropometric strata may therefore clarify how lipid-derived indices vary across contexts.

This study aimed to examine whether the interpretive behavior of routine composite cardiometabolic indices varies according to central adiposity and the evaluated three-variant triglyceride-weighted wGRS in a Mexican adult cohort. We evaluated the joint structural behavior of AIP and TyG, assessed the WHtR × wGRS interaction in index expression, and characterized the distributional heterogeneity of standardized AIP within WHtR strata by sex. By integrating correlation architecture, interaction modeling, and distribution-aware visualization, we investigated whether wGRS-related separation persisted at higher levels of central adiposity and whether anthropometric stratification modified metabolic overlap. This approach clarifies how contextual variability shapes the interpretation of the composite cardiometabolic indices.

## 2 Materials and methods

### 2.1 Study design and participants

A cross-sectional analytical design was applied to evaluate the behavior and interpretability of lipid- and glucose-derived cardiometabolic indices across the central adiposity strata in Mexican adults. Participants were recruited at the Nutrition Clinic of Universidad Iberoamericana Ciudad de México during their first nutritional counseling visit. The final analytical sample comprised 396 individuals (306 women and 90 men), aged 19–65 years.

**Figure 1:**
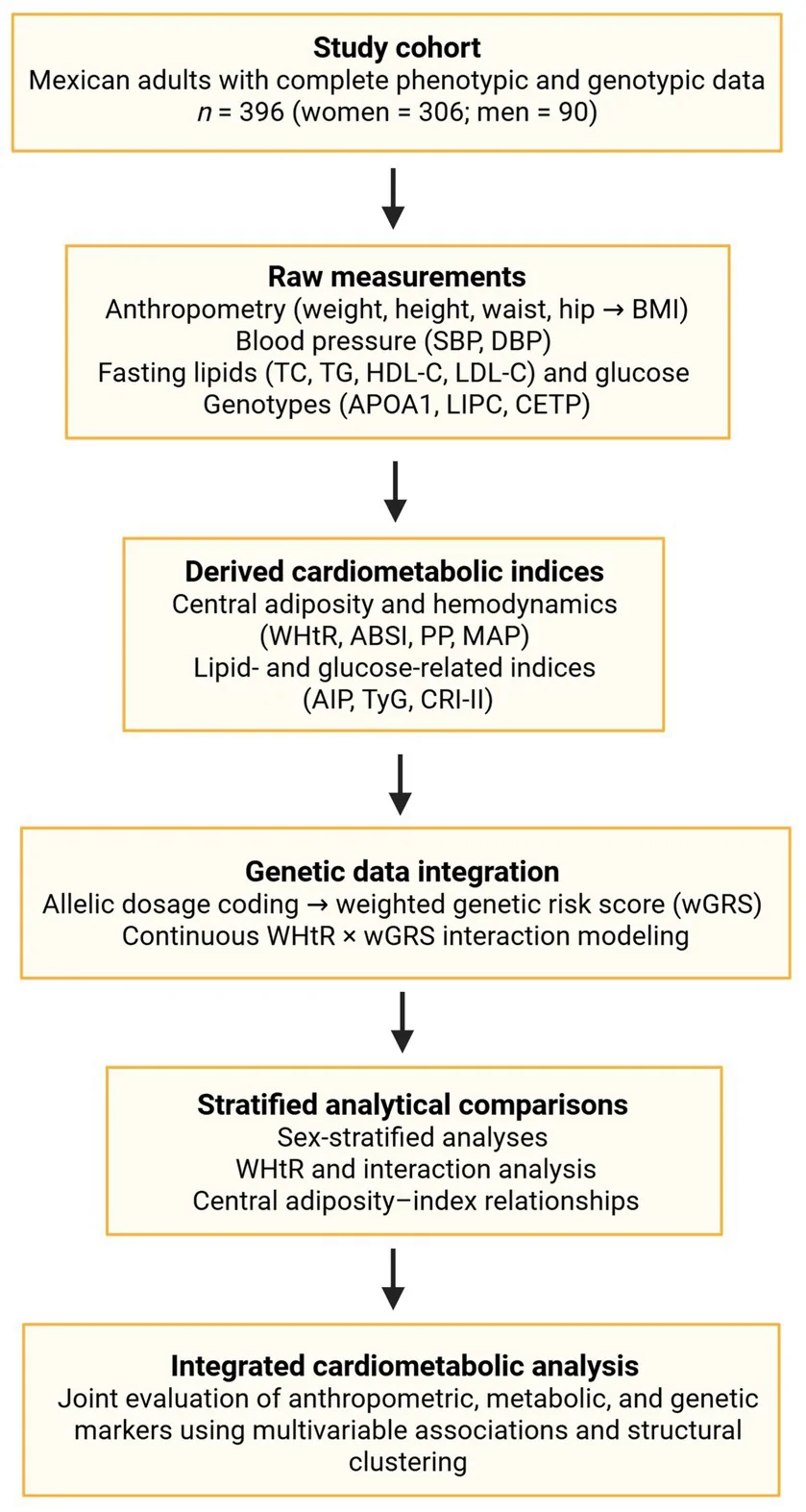
Study design and analytical workflow. Raw anthropometric and biochemical data were transformed into derived cardiometabolic indices, while genotype data from three variants were integrated into the wGRS for sex-stratified analyses in Mexican adults.

All participants completed a structured questionnaire covering medical history, physical activity, dietary habits, and self-reported alcohol consumption, recorded as no or yes. Individuals with incomplete anthropometric, biochemical, or genetic data were excluded from the analyses. The overall analytical workflow integrating anthropometric, metabolic, and genetic variables is summarized in Figure 1.

### 2.2 Inclusion and exclusion criteria

The eligibility criteria were defined to minimize confounding by conditions known to affect lipid and glucose metabolism (Supplementary Table S1). Individuals with obesity (body mass index [BMI] ≥ 30 kg/m^2^) were included in the absence of autoimmune disease, cancer, eating disorders, pregnancy or lactation, acute illness, and changes in dosage or initiation of lipid-lowering therapy within the 6 months preceding enrollment. The participants on stable maintenance therapy were retained. The same criteria were applied to normal-weight participants (BMI 18.5–24.9 kg/m^2^). The use of lipid-lowering, antihypertensive, and glucose-lowering medication was recorded for all the participants. In women, menopausal status and current use of hormonal therapy or contraceptives were documented.

### 2.3 Ethical approval

The study was conducted in accordance with the Declaration of Helsinki and approved by the Comité de Ética en Investigación de la Universidad Iberoamericana Ciudad de México (protocol code 043/2020; approved on January 4, 2020). Written informed consent was obtained from all participants prior to data collection.

### 2.4 Anthropometric and blood pressure measurements

Anthropometric measurements were obtained by trained personnel following standardized clinical procedures (15, 16). Body weight and height were measured with the participants wearing light clothing and no shoes. BMI was calculated as kg/m^2^.

Waist circumference was measured using a non-elastic tape at the midpoint between the lower margin of the last palpable rib and the iliac crest, with participants standing upright at the end of normal expiration (17). The waist-to-height ratio (WHtR) was calculated as the waist circumference divided by height (cm/cm). The Body Shape Index (ABSI) and weight-adjusted waist index (WWI) were derived according to previously published definitions (15).

Systolic and diastolic blood pressure were measured under standardized clinical conditions. Pulse pressure (PP) and mean arterial pressure (MAP) were calculated using Equations 1 and 2 respectively:

$$\mathrm{PP}=P_{s}-P_{d}$$ (1)

$$\mathrm{MAP}=P_{d}+\frac{P_{s}-P_{d}}{3}$$ (2)

where *P*_s_ represents systolic blood pressure (mmHg), and *P*_d_ represents diastolic blood pressure (mmHg), as commonly applied in population-based cardiometabolic studies (1).

### 2.5 Blood sampling and biochemical analysis

Blood samples were obtained during routine clinical visits after a protocol-required overnight fast of 8–12 h. Fasting status was verified and recorded for all participants at the time of blood collection. Blood sampling was performed during a single clinical visit, and the time of collection was documented. Serum samples were stored at −60 °C until further analysis.

Serum concentrations of total cholesterol, triglycerides (TG), high-density lipoprotein cholesterol (HDL-C), low-density lipoprotein cholesterol (LDL-C), very-low-density lipoprotein cholesterol (VLDL-C), and fasting glucose were determined using a DRI-CHEM NX500i analyzer (Fujifilm, Tokyo, Japan) following the manufacturer’s instructions (18). All biochemical variables are expressed in mg/dL.

### 2.6 Derived metabolic indices

The atherogenic index of plasma (AIP) was calculated as previously described (19) using Equation 3:

$$\mathrm{AIP}=\log_{10}(\frac{\mathrm{TG}}{\mathrm{HDL}-C})$$ (3)

where TG represents triglycerides (mg/dL), and HDL-C represents high-density lipoprotein cholesterol (mg/dL).

The triglyceride–glucose (TyG) index was calculated as an integrated lipid–glucose marker commonly used as a surrogate for insulin resistance (1, 20) according to Equation 4:

$$\mathrm{TyG}=\ln(\frac{\mathrm{TG}\times \text{Glucose}}{2})$$ (4)

where TG and Glucose are expressed in mg/dL.

The Castelli Risk Index II (CRI-II), which reflects the balance between atherogenic and antiatherogenic cholesterol fractions (21, 22), was calculated using Equation 5:

$$\mathrm{CRI}-\mathrm{II}=\frac{\mathrm{LDL}-C}{\mathrm{HDL}-C}$$ (5)

where LDL-C and HDL-C are expressed in mg/dL.

### 2.7 Genetic risk score

A weighted genetic risk score (wGRS) was constructed using three triglyceride-associated variants (23, 24). The score was calculated using Equation 6:

$$\text{wGRS}=\sum_{i=1}^{3}(G_{i}\times \beta_{i})$$ (6)

where $G_{i}$ represents the effect-allele count (0, 1, or 2) for SNP i , and $\beta_{i}$ represents the corresponding per-allele triglyceride effect estimate obtained from the Global Lipids Genetics Consortium (GLGC) 2021 multi-ancestry summary statistics for triglycerides (25). Effect alleles were aligned with the GLGC coding reported in Supplementary Table S2.

Genotyping quality was assessed using standard procedures. The genotype call rate was 100% for all SNPs, and the Hardy–Weinberg equilibrium was confirmed in the overall sample (Supplementary Table S3). Genotype data consisted of the three wGRS variants and did not provide the genomic marker coverage required to estimate individual ancestry proportions or genetic ancestry principal components.

### 2.8 Stratification strategy

Participants were stratified by sex and WHtR tertiles defined according to the empirical distribution of the study population: T1 ≤ 0.47, T2 = 0.48–0.61, and T3 ≥ 0.62, consistent with prior population-based applications (26, 27).

These WHtR strata were used for descriptive and distributional analyses and were not intended as clinical cutoff values. Formal effect modification was assessed using continuous WHtR and wGRS terms in regression models, with standardized estimates used for interpretation.

### 2.9 Statistical analysis

Statistical analyses were performed using OriginPro 2025b (v10.2.5.212; OriginLab Corporation, Northampton, MA, USA). Continuous variables are presented as mean ± standard deviation or median [interquartile range] as appropriate. Normality was assessed using the Shapiro–Wilk test.

**Figure 2:**
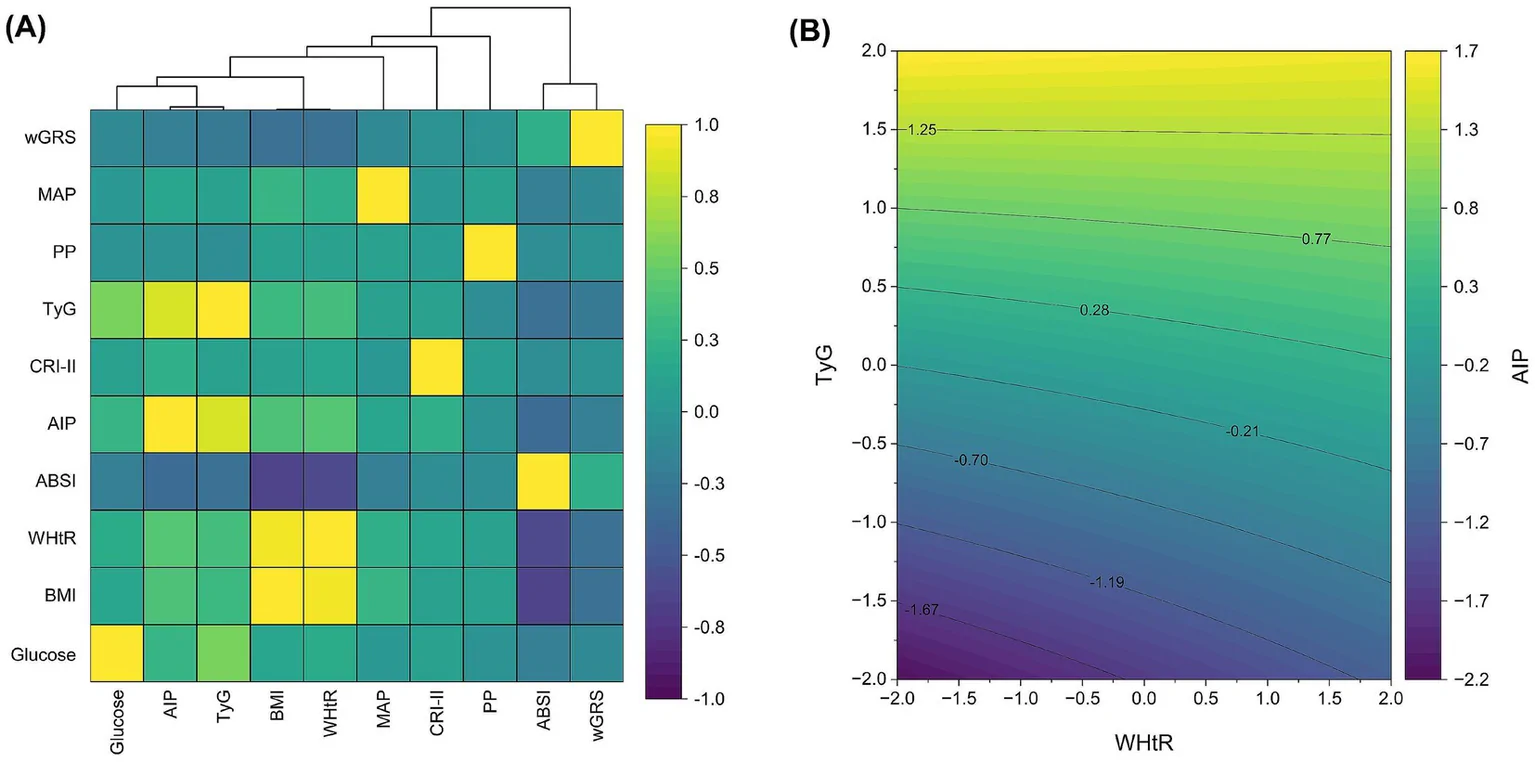
Age- and sex-adjusted structure of cardiometabolic relationships. **(A)** Heatmap showing Spearman correlation coefficients (*ρ*) and hierarchical clustering among cardiometabolic indicators expressed as *z*-scores. **(B)** Continuous response surface of AIP (*z*-score) across TyG and WHtR (both *z*-scores), with contour lines indicating equal AIP values and a steeper gradient along the TyG axis within the evaluated range.

Between-group comparisons were performed using the Student’s *t*-test or Mann–Whitney U test, and comparisons across WHtR tertiles were conducted using one-way ANOVA or Kruskal–Wallis tests, depending on distributional assumptions. When ANOVA indicated significant differences, pairwise comparisons were performed using Tukey’s honestly significant difference test. Associations were evaluated using Spearman correlation coefficients computed on residuals obtained after linear adjustment for age and sex (28, 29). The response surface shown in Figure 2B was generated from the predicted values of the multivariable linear regression model, evaluated over a regular grid spanning the observed ranges of WHtR and TyG.

Effect modification was assessed using linear regression models including a multiplicative interaction term between WHtR and wGRS, as specified in Equation 7, with the interaction magnitude interpreted from standardized estimates to reduce scale dependency between predictors:

$$\text{Outcome}=\beta_{0}+\beta_{1}WH_{t}R+\beta_{2}\text{wGRS}+\beta_{3}(WH_{t}R\times \text{wGRS})+\varepsilon$$ (7)

where WHtR denotes the waist-to-height ratio expressed as a continuous variable. All regression models were additionally adjusted for age, sex (where applicable), fasting status, reported cardiometabolic medication use during the clinical evaluation (yes/no status), and self-reported alcohol consumption (no/yes). Adjusted means and standard errors (SE) reported in the stratified analyses were obtained from the corresponding multivariable regression models.

Collinearity diagnostics were evaluated prior to model interpretation (5). Minimum detectable effects (MDEs) were calculated for the sex-stratified WHtR × wGRS interaction models at 80% power and a two-sided *α* of 0.05 (Supplementary Table S9). All inferential analyses involving the wGRS were interpreted as exploratory. Analyses were conducted on complete cases without data imputation because all 396 participants had complete data for the variables included in the multivariable models, including alcohol consumption. All statistical tests were two-sided, and 𝑝 < 0.05 was considered statistically significant.

## 3 Results and discussion

### 3.1 Baseline cardiometabolic overview

Table 1 presents the baseline cardiometabolic characteristics of the Mexican adult cohort stratified by sex, and Table 2 summarizes these variables across WHtR tertiles. In sex-stratified comparisons, men exhibited higher WHtR and MAP than women, whereas mean AIP, TyG, and fasting glucose values did not differ significantly between sexes. Despite their higher WHtR, men had lower ABSI values (𝑝 < 0.001), indicating that these anthropometric indices capture distinct aspects of body composition. When participants were stratified by WHtR tertiles (*n* = 132 per group), mean WHtR increased from 0.42 to 0.70. Across the same tertiles, mean age increased from 25.71 to 42.67 years and mean BMI from 21.77 to 40.79 kg/m^2^ (𝑝 < 0.001). The age gradient across tertiles could also have contributed to the observed metabolic differences through age-related shifts in body composition. AIP, TyG, fasting glucose, and MAP were higher in the upper WHtR tertiles than in T1, whereas mean wGRS decreased across tertiles (Δ = −0.05; 𝑝 < 0.001). For AIP, Tukey *post hoc* comparisons showed that the observed difference was driven by separation of T1 from T2 and T3. Because T2 and T3 did not differ (𝑝 = 0.917), the result was consistent with a T1-versus-upper-tertiles contrast rather than a continuous gradient across all three groups. Compared with the limited differences observed between sexes, stratification by WHtR revealed consistent variation across anthropometric, metabolic, and hemodynamic measures. Additionally, the proportion of participants receiving antihypertensive and glucose-lowering treatment was highest in the upper WHtR tertile, which corresponded to the tertile with the highest values of metabolic and blood pressure markers.

**Table 1:** Baseline cardiometabolic characteristics of the study population stratified by sex.

Variable	Women (*n* = 306)	Men (*n* = 90)	𝑝-value
Age (years)	35.98 ± 14.06	36.54 ± 13.48	0.732
Glucose (mg/dL)	96.16 ± 33.43	92.17 ± 15.16	0.110
BMI (kg/m^2^)	31.43 ± 9.26	32.74 ± 8.56	0.212
WHtR	0.56 ± 0.13	0.59 ± 0.12	0.036
ABSI	0.09 ± 0.01	0.08 ± 0.01	<0.001
AIP	0.46 ± 0.28	0.47 ± 0.25	0.759
CRI-II	2.07 ± 0.85	2.11 ± 0.73	0.694
TyG index	8.68 ± 0.70	8.62 ± 0.54	0.413
PP (mmHg)	39.64 ± 10.07	42.32 ± 10.55	0.042
MAP (mmHg)	90.83 ± 11.03	98.20 ± 12.09	<0.001
wGRS	0.10 ± 0.07	0.11 ± 0.07	0.663

Data are mean ± standard deviation (SD). 𝑝-values were calculated using Student’s *t*-test. BMI, body mass index; WHtR, waist-to-height ratio; ABSI, A Body Shape Index; AIP, atherogenic index of plasma; CRI-II, Castelli Risk Index II; TyG, triglyceride–glucose index; PP, pulse pressure; MAP, mean arterial pressure; wGRS, weighted genetic risk score.

**Table 2:** Anthropometric, metabolic, and hemodynamic variables across WHtR tertiles.

Variable	T1 (*n* = 132)	T2 (*n* = 132)	T3 (*n* = 132)	𝑝-value
Age (years)	25.71 ± 9.51 ^a^	39.95 ± 13.28 ^b^	42.67 ± 12.26 ^b^	<0.001
Glucose (mg/dL)	86.82 ± 30.45 ^**a**^	99.29 ± 27.46 ^**b**^	99.65 ± 31.26 ^**b**^	<0.001
BMI (kg/m^2^)	21.77 ± 1.59 ^a^	32.62 ± 3.80 ^b^	40.79 ± 7.06 ^c^	<0.001
WHtR	0.42 ± 0.03 ^**a**^	0.58 ± 0.04 ^**b**^	0.70 ± 0.06 ^**c**^	<0.001
ABSI	0.09 ± 0.01 ^a^	0.09 ± 0.00 ^b^	0.08 ± 0.01 ^c^	<0.001
AIP	0.25 ± 0.23 ^a^	0.56 ± 0.22 ^b^	0.58 ± 0.22 ^b^	<0.001
CRI-II	1.88 ± 0.75 ^a^	2.14 ± 0.76 ^b^	2.21 ± 0.91 ^b^	0.002
TyG index	8.19 ± 0.57 ^a^	8.92 ± 0.57 ^b^	8.89 ± 0.58 ^b^	<0.001
PP (mmHg)	36.66 ± 8.40 ^a^	41.52 ± 14.57 ^b^	42.57 ± 10.76 ^b^	<0.001
MAP (mmHg)	85.67 ± 7.79 ^a^	93.96 ± 11.78 ^b^	97.89 ± 11.56 ^c^	<0.001
wGRS	0.13 ± 0.06 ^a^	0.10 ± 0.07 ^b^	0.08 ± 0.06 ^c^	<0.001

Data are mean ± SD. One-way ANOVA was used. Within each row, different superscript letters indicate significant differences (Tukey test, 𝑝 < 0.05). BMI, body mass index; WHtR, waist-to-height ratio; ABSI, A Body Shape Index; AIP, atherogenic index of plasma; CRI-II, Castelli Risk Index II; TyG, triglyceride–glucose index; PP, pulse pressure; MAP, mean arterial pressure; wGRS, weighted genetic risk score.

Previous research has examined WHtR in relation to cardiometabolic risk, mainly as a tool to identify individuals with hypertension and clustered metabolic abnormalities. Gu et al. (30) reported that WHtR showed a similar AUC to BMI and waist circumference for detecting multiple metabolic risk factors in Chinese older adults (AUC ≈ 0.688 in men). In a prospective analysis from the China Health and Nutrition Survey (CHNS), Wang et al. (26) found that higher WHtR was associated with incident hypertension, which is consistent with the higher MAP observed in the upper WHtR tertile in the present cohort. Using computed tomography (CT) to quantify visceral fat area, Hussain et al. (31) showed that the highest tertile of visceral adiposity had a greater prevalence of metabolic syndrome and hypertension than BMI-defined categories, supporting the relevance of central fat distribution beyond overall body mass. In a clinical study using the Matsuda index, Lechner et al. (32) reported an inverse correlation between WHtR and insulin sensitivity (*r* ≈ −0.458, 𝑝 < 0.001). In the same study, WHtR showed good discrimination for insulin resistance (AUC ≈ 0.80), suggesting that it captures metabolic variation not fully reflected by fasting glucose alone. While these studies emphasize prediction and risk detection, the present findings show that higher WHtR within a single cohort is accompanied by higher AIP, TyG, fasting glucose, and MAP values, with the clearest contrasts observed between the lowest and upper tertiles.

The higher AIP and TyG values in the upper WHtR tertiles reflect metabolic changes accompanying greater central adiposity. As WHtR increases, greater free fatty acid delivery to the liver promotes VLDL production and may reduce HDL concentrations (33), directly influencing the components of both indices. Within this setting, the attenuation of wGRS-related differences indicates that AIP separation associated with the evaluated three-variant score was smaller at higher WHtR levels. The decrease in mean wGRS across WHtR tertiles describes the distribution of this score within the cohort rather than overall triglyceride-related genetic susceptibility. This pattern is compatible with a context-dependent association between the evaluated wGRS and AIP across WHtR levels in this cohort. Because both AIP and TyG depend on circulating triglycerides, their simultaneous rise across adiposity strata indicates that part of their variation may arise from a shared biochemical pathway. This issue is particularly relevant in Mexican adults, among whom central adiposity and hypertriglyceridemia are common, and composite indices are frequently interpreted independently in clinical and nutritional practice. Given the cross-sectional design and the use of WHtR as a proxy for visceral fat, these findings describe associations rather than temporal effects. Longitudinal studies are needed to clarify how changes in adiposity influence the behavior of these indices over time.

### 3.2 Interrelationships among cardiometabolic indices

Figure 2 presents Spearman correlation coefficients calculated from residuals adjusted for age and sex, together with the corresponding response surface for AIP, TyG, and WHtR (*n* = 396). In the correlation matrix (Figure 2A), BMI and WHtR showed a strong positive association (*ρ* = 0.93), and AIP and TyG were also strongly correlated (*ρ* = 0.87). AIP showed moderate correlations with WHtR (*ρ* = 0.42) and BMI (*ρ* = 0.41), whereas TyG correlated with glucose (*ρ* = 0.57) and more weakly with WHtR (*ρ* = 0.36) and BMI (*ρ* = 0.33). ABSI was inversely correlated with BMI (*ρ* = −0.63) and WHtR (*ρ* = −0.62), and wGRS showed small negative correlations with both AIP and TyG. In the response surface (Figure 2B), standardized AIP values increased primarily along the TyG dimension, while variation across WHtR at similar TyG values was smaller. Contour values ranged from −1.67 to 1.25, and their near-horizontal configuration indicates that changes in AIP within the evaluated range were largely aligned with changes in TyG rather than with WHtR. This structural configuration provides a basis for evaluating whether AIP and TyG convey distinct information or largely reflect shared triglyceride-driven variation within comparable adiposity contexts.

Previous studies have reported modest incremental discrimination when triglyceride-derived indices are combined. Chen et al. (5) showed that TyG-AIP improved CVD prediction only slightly compared to TyG or AIP alone, with limited gains in AUC and reclassification. Similarly, Shi et al. (28) observed that lipid-based indices did not consistently outperform structural adiposity measures for mortality in metabolic syndrome. Metabolomic analyses further indicate partial biological overlap among non-allometric adiposity indices, with median shared metabolite proportions of approximately 42%, whereas allometric measures display more distinct signatures (12). Imaging analyses also demonstrated that correlated visceral adiposity metrics (*r* ≈ 0.68) can nonetheless reflect differential associations with lipid, glycemic, and inflammatory markers (3). In the present data, the concentration of variance within the triglyceride-derived pair is consistent with reports of partial overlap and limited additive discrimination.

Figure 2B shows a TyG-dominated gradient with near-horizontal AIP contours, and Figure 2A reports a strong AIP–TyG correlation (*ρ* ≈ 0.87). This configuration indicates that much of the variation between individuals is concentrated along a triglyceride-related axis, which may help explain why the apparent differences between AIP and TyG are often small in practice. Greater central adiposity is associated with increased visceral lipolysis, which may elevate hepatic free fatty acid delivery and VLDL-triglyceride production (3). Therefore, higher circulating triglycerides influence both composite indices: TyG incorporates triglycerides with fasting glucose, whereas AIP reflects the triglyceride-to-HDL-C ratio. Triglyceride enrichment may also coincide with a relative reduction in HDL levels, as observed in atherogenic dyslipidemia (5). Both indices include triglycerides by construction, and their strong association (*ρ* ≈ 0.87) was attenuated after adjustment for this component (*r* = −0.118; Supplementary Table S4). This attenuation indicates that much of their shared variation is triglyceride-driven, so close agreement between AIP and TyG may reflect shared structure rather than fully independent metabolic information.

These findings indicate that at higher levels of central adiposity, AIP adds limited additional information beyond TyG. They also suggest that discriminative contrast depends more on multivariate organization than on small absolute differences alone. The strength of this dependence may vary with the distribution of adiposity in the sample. In practical assessments, agreement between AIP and TyG may reflect structural coupling, consistent with reports of limited incremental discrimination when both indices are combined (5). Analytical models that explicitly control for triglycerides may help separate compositional overlap from residual biological co-variation and evaluate whether index dependence persists across broader anthropometric contexts.

### 3.3 wGRS–central adiposity interaction

**Figure 3:**
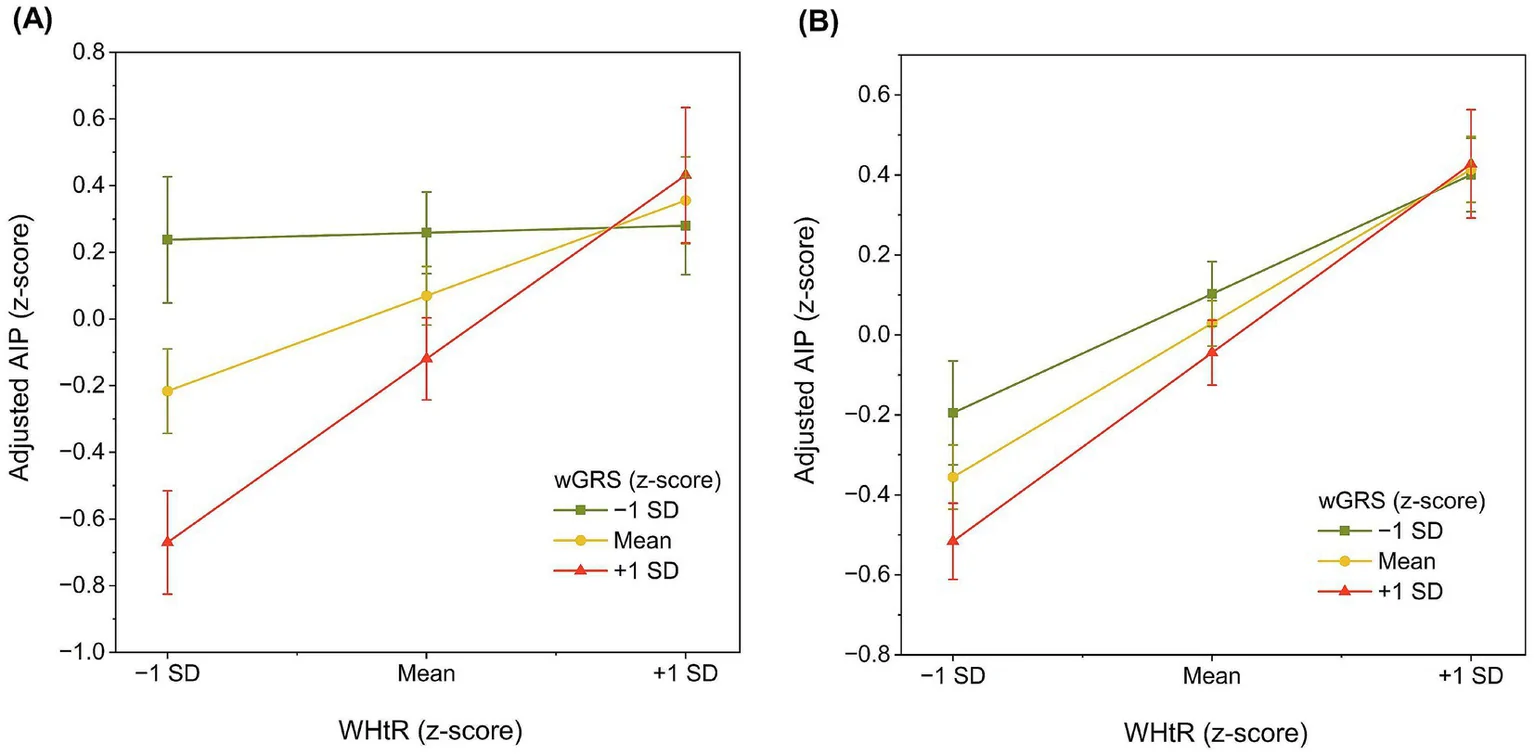
Adjusted AIP estimates across standardized WHtR and wGRS values by sex **(A)** Men and **(B)** women. WHtR and wGRS are shown at −1 SD, the mean, and +1 SD. Error bars represent standard errors (SE); corresponding 95% confidence intervals are reported in Supplementary Table S10. Sex-stratified wGRS estimates were considered exploratory.

Figure 3 extends the preceding analysis of triglyceride-related overlap by showing how adjusted AIP estimates varied across standardized WHtR and wGRS values in men and women. In men, the absolute contrast between wGRS −1 SD and +1 SD decreased from 0.908 AIP z-score units at WHtR −1 SD to 0.152 units at WHtR +1 SD. The corresponding contrast in women decreased from 0.321 to 0.027 units (Supplementary Table S10). At lower WHtR, point estimates were lower at higher wGRS, whereas at higher WHtR they converged and reversed in ordering in both sexes. This narrowing indicates that the wGRS-related separation in predicted AIP became less distinct as WHtR increased. The same difference in wGRS did not correspond to a constant AIP contrast across the observed adiposity range. Comparable AIP estimates may arise from different combinations of WHtR and wGRS within this cohort. Central adiposity provides additional context for interpreting between-person differences in AIP.

The adjusted WHtR × wGRS coefficient was positive in the pooled analysis (*β* = 0.0449, 95% CI 0.0199–0.0700, 𝑝 < 0.001; Supplementary Table S5); the fitted wGRS–AIP slope varied across WHtR. Sex-stratified estimates were treated as exploratory. In men (*n* = 90), *β* was 0.2649 (95% CI 0.1013–0.4284, 𝑝 = 0.002), compared with an MDE of 0.2331. In women (*n* = 306), *β* was 0.0870 (95% CI −0.0257–0.1998, 𝑝 = 0.130), compared with an MDE of 0.1610 (Supplementary Table S9). In a separate women-only model additionally adjusted for menopausal status, hormonal therapy, and contraceptive use, the coefficient was positive (*β* = 0.0365, 95% CI 0.0065–0.0665, 𝑝 = 0.017; Supplementary Table S6). The estimated coefficient exceeded the MDE in men but not in women; however, this difference does not establish that the interaction is restricted to men. The positive estimate in the reproductive-covariate model was compatible with an association in women, although its magnitude remained uncertain within this cohort.

Previous studies have reported adiposity-dependent genetic associations with individual lipid traits. Ahmad et al. (6) observed stronger TG-wGRS slopes for triglycerides and triglyceride-rich lipoproteins at higher BMI and waist circumference, while Esteve-Luque et al. (7) reported a stronger GRS–TG association in individuals with obesity. Hernández-Guerrero et al. (8) identified a GRS × waist-to-hip ratio interaction for LDL-C. Chermon and Birk (34) reported less favorable lipid profiles in groups with higher polygenic risk, especially in obesity. These reports linked adiposity with variation in lipid-related genetic associations, although their outcomes and scores differed. In this cohort, wGRS-related AIP separation was greatest at lower WHtR and narrowed as WHtR increased. This difference may partly arise because AIP integrates triglyceride and HDL-C variation, whereas the cited interaction studies used individual lipid endpoints. An association evident in a single lipid may appear differently once incorporated into a composite index. The present findings extend this comparison to AIP without equating it with individual lipid outcomes.

The component models support a triglyceride-related contribution to this response. Supplementary Table S4 shows a positive WHtR × wGRS coefficient for standardized triglycerides (*β* = 0.1310, 95% CI 0.0319–0.2302, 𝑝 = 0.010). For HDL-C, the coefficient was −0.0783 (95% CI −0.1794–0.0227, 𝑝 = 0.128), and its confidence interval included zero. The interaction was also positive when TyG was the outcome (*β* = 0.1026, 95% CI 0.0401–0.1652, 𝑝 = 0.001; Supplementary Table S7). The concordant AIP and TyG interactions may partly reflect their shared triglyceride component and provide related, but not independent, evidence. This distinction is relevant when both indices are used in the same metabolic assessment. Across the observed WHtR range, the association of the score with both indices differed by adiposity level. The convergence at higher WHtR indicates smaller wGRS-related separation in predicted values without implying altered effects of underlying variants. Interpreting either index with WHtR and sex may provide a more specific description of the observed metabolic state.

**Figure 4:**
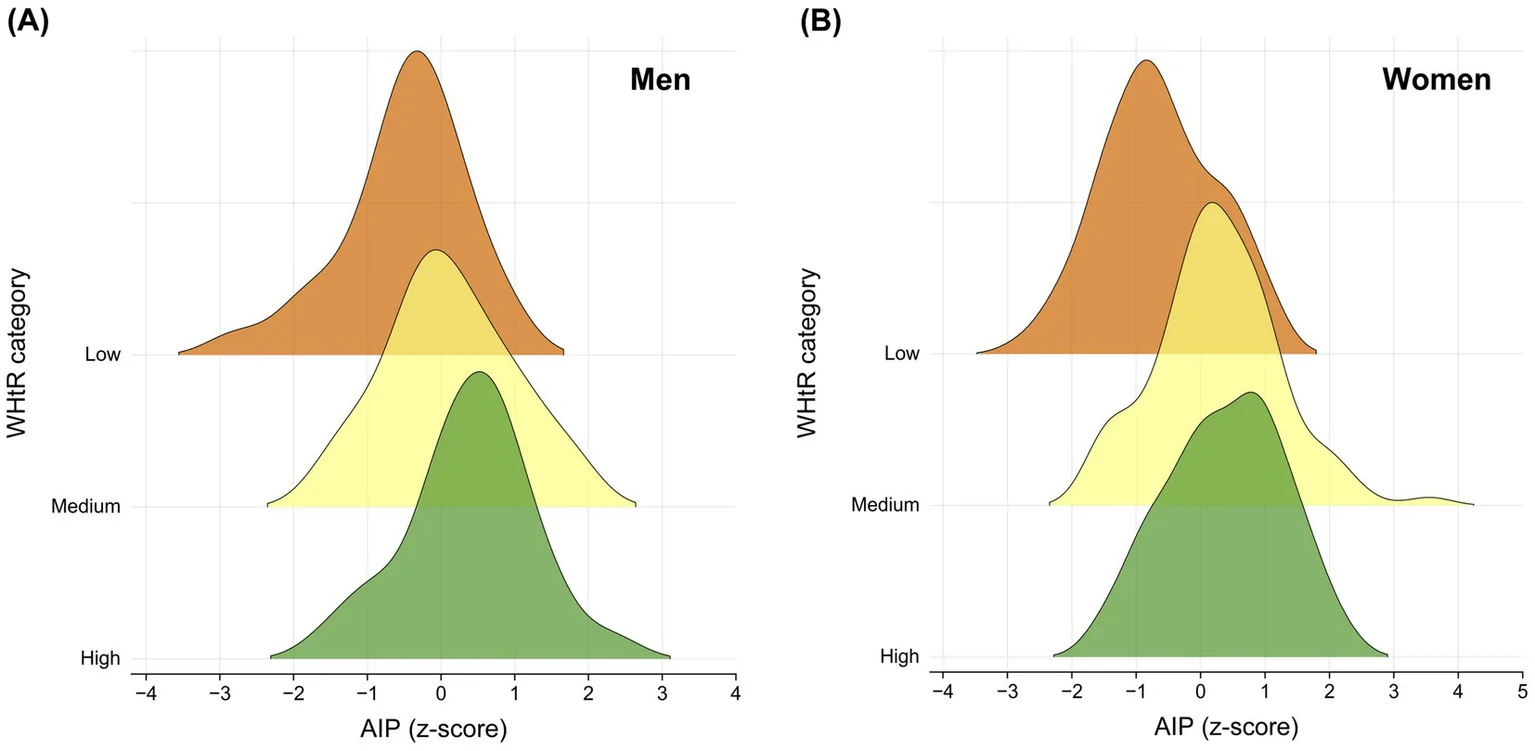
Sex-stratified distributions of the atherogenic index of plasma across waist-to-height ratio categories. **(A)** Men and **(B)** women. Ridgeline density plots show standardized AIP (z-scores) across low, medium, and high WHtR categories within each sex. These sex-stratified distributions were considered exploratory.

Predicted AIP values converged across wGRS levels at higher WHtR, indicating that comparable index values may occur despite different score levels. This finding suggests that AIP alone cannot indicate how much of an observed value corresponds to score-related or adiposity-related variation. The results support interpreting AIP in relation to WHtR and sex, but they do not establish improved predictive performance or added clinical utility. Sex-specific estimates remained exploratory, and self-reported alcohol consumption was included as a categorical covariate. Variation in drinking intensity, physical activity, and dietary fat intake may still contribute to residual confounding. Ancestry-resolved analyses could clarify whether population structure contributes to the observed wGRS associations and test score portability across admixed populations (35, 36). These wGRS-related findings remain exploratory and are restricted to the specific three-variant score and cohort evaluated. Comparable adjusted means may still conceal substantial individual variability, as reflected by the sex-specific AIP distributions across WHtR categories in Figure 4.

### 3.4 Metabolic heterogeneity patterns

Figure 4 presents the sex-stratified distributions of standardized AIP (z-scores) across the WHtR categories. Median AIP in men (Figure 4A) increased from −0.35 in the low-WHtR group (*n* = 30) to 0.04 in the medium group (*n* = 32) and 0.44 in the high group (*n* = 28). Median values in women (Figure 4B) shifted from −0.73 (low, *n* = 102) to 0.21 (medium, *n* = 100) and 0.38 (high, *n* = 104). Kruskal–Wallis tests indicated significant differences across WHtR categories within each sex (𝑝 < 0.001), primarily reflecting separation of the low category. Brown–Forsythe tests indicated comparable dispersion across strata (𝑝 ≥ 0.84), while the medium and high categories retained partially overlapping value ranges in both sexes. In women, the medium-WHtR group reached a higher maximum AIP (3.55) than the high group (2.26), with five versus two observations exceeding 2.0 z-score units. These exploratory findings reflect a directional shift in central tendency, with the clearest separation involving the low category, while variability persisted within WHtR categories.

Previous studies have shown that individuals within the same anthropometric category may not share the same metabolic risk profile. Papagiannopoulos et al. (12) reported partially distinct metabolic signatures across adiposity indices, and the dispersion of AIP in Figures 4A,B demonstrates comparable within-stratum breadth. Jia et al. (9) identified multiple metabolic positions within a single BMI range using nonlinear dimensionality reduction, indicating intra-category heterogeneity along a lipid-derived dimension. Chami et al. (10) described the genetic uncoupling between adiposity and lipid traits, and Agrawal et al. (11) reported anatomical variability within BMI strata. Collectively, these reports indicate that anthropometric similarity does not ensure metabolic uniformity. In this study, similar within-stratum variability was observed across WHtR categories. Persistent within-stratum variability may reflect biological processes that influence lipid profiles under comparable anthropometric conditions. Further analyses are needed to determine whether this variability differs by sex across intermediate and higher WHtR ranges.

Xue et al. (37) described cardiometabolic heterogeneity within central obesity phenotypes, a pattern compatible with the distributional breadth observed in this dataset. Klein et al. (3) reported depot-level differences in visceral adiposity associated with lipid and glycemic profiles, suggesting that individuals with comparable WHtR may differ in their lipid-handling characteristics. AIP, defined as log(TG/HDL-C), responds to coordinated shifts in triglyceride and HDL-C levels that can yield broad index ranges within shared anthropometric categories (2). Li et al. (4) documented variability across triglyceride-related indices in metabolically diverse samples, further indicating the sensitivity of lipid-derived markers to underlying metabolic variations. Imaging studies have also described sex differences in fat distribution, which may be related to the broader upper range observed in women (Figure 4B). These observations are compatible with heterogeneous lipid configurations under comparable levels of central adiposity and provide a physiological context for the dispersion observed in Figure 4.

Figure 4 supports evaluating composite lipid indices beyond central tendency when heterogeneity is expressed as an upper-quantile spread within anthropometric categories. Kernel density visualization enabled inspection of the distribution shape across sex-specific WHtR strata without imposing parametric assumptions. This descriptive strategy characterized overlap and dispersion, but it did not test whether the WHtR–AIP relationship changes nonlinearly along the continuous WHtR range. Recent restricted cubic spline analyses have identified inflection points in AIP under changing metabolic conditions, indicating that variation in this index may follow threshold behavior rather than strictly proportional change (38). In the present dataset, the clearer separation of the low category from the upper tertiles is consistent with that possibility, but no spline-based or other continuous nonlinear model was fitted. Therefore, the existence of a specific WHtR threshold at which AIP changes accelerate cannot be established in this study. Sex-stratified quantile regression models showed that the WHtR × wGRS interaction remained significant at the 0.75 quantile in men (*β* = 0.312, 𝑝 = 0.002) and women (*β* = 0.166, 𝑝 = 0.028). At the 0.90 quantile, the interaction was not statistically significant in men (*β* = 0.148, 𝑝 = 0.070) or women (*β* = 0.037, 𝑝 = 0.638; Supplementary Table S8). These models provide a formal evaluation of upper-quantile behavior beyond descriptive density visualization. Such analytical refinement may improve the structural evaluation of lipid-derived indices in heterogeneous populations, while maintaining direct comparability with the empirical distributions shown in Figure 4.

## 4 Conclusion

This study showed that the central adiposity context is associated with differences in the structural and distributional behavior of triglyceride- and glucose-derived composite indices in Mexican adults. AIP was higher in T2 and T3 than in T1 (0.56 and 0.58 vs. 0.25), whereas the difference between T2 and T3 was minimal. The observed contrast therefore reflected separation of T1 from the upper two tertiles, not a continuous gradient across all three groups. TyG was likewise higher in the upper WHtR tertiles than in T1, reinforcing the upward displacement of this composite marker with greater central adiposity. Exploratory sex-stratified predictions from the three-variant wGRS models showed that the absolute AIP difference between wGRS −1 SD and +1 SD narrowed as WHtR increased from −1 SD to +1 SD. The contrast declined from 0.91 to 0.15 z-score units in men and from 0.32 to 0.03 in women. This study evaluated structural coupling, the WHtR × wGRS interaction, and within-stratum dispersion across the adiposity gradient. These conclusions are limited to the cross-sectional, clinic-based design and the WHtR-defined adiposity strata evaluated in this study. The findings therefore describe associations within this setting and should not be interpreted as evidence of longitudinal risk prediction or causal inference. These findings suggest that central adiposity is associated with variations in the interpretive range and discriminatory capacity of composite lipid indices, supporting a more context-aware approach to metabolic profiling. Future longitudinal studies incorporating ancestry-resolved genetic data could assess whether within-stratum dispersion and wGRS-related contrast change over time.

## Data Availability

The datasets presented in this article are not readily available because they contain individual-level clinical and genetic data, and access is restricted to protect participant confidentiality. Requests to access the datasets should be directed to César Hernández-Guerrero (cesar.hernandez@ibero.mx).
