# Supplementary material for: Central adiposity and sex influence lipid and glucose indices in Mexican adults: implications for metabolic risk assessment
Source: Front Nutr. 2026 Aug 26;13:1834724. doi: 10.3389/fnut.2026.1834724 (PMC13553771; doi:10.3389/fnut.2026.1834724)
Supplement: Supplementary file 1 [file Data_Sheet_1.docx]

Supplementary Material

**Supplementary Figure 1.** Age- and sex-adjusted Spearman correlation matrix. Values represent Spearman correlation coefficients (*ρ*) calculated from age- and sex-adjusted residuals. Hierarchical clustering is displayed above the matrix.

**Supplementary Table 1.** Participant inclusion and exclusion criteria.

| Inclusion criteria | Exclusion criteria |
| --- | --- |
| BMI ≥ 30 kg/m^2^ (obesity group) | Autoimmune disease |
| BMI 18.5–24.9 kg/m^2^ (normal-weight group) | Active cancer |
|  | Eating disorders |
|  | Pregnancy or lactation |
|  | Acute illness at the time of evaluation |
|  | Unstable or recent initiation of lipid-lowering drugs |

**Supplementary Table 2.** Externally weighted SNPs used to construct the triglyceride-weighted genetic risk score (wGRS).

| Gene | SNP | Effect allele | *β* (logTG) | SE | 𝑝-value |
| --- | --- | --- | --- | --- | --- |
| APOA1 | rs670 | C | 0.024 | 0.002 | < 0.001 |
| LIPC | rs1800588 | C | 0.042 | 0.002 | < 0.001 |
| CETP | rs708272 | G | 0.002 | 0.000 | 0.048 |

Effect alleles indicate the coding used in the present analysis. Genotypes were coded as 0, 1, or 2 according to the effect-allele count. The β coefficients were obtained from the GLGC 2021 multi-ancestry summary statistics for triglycerides.

**Supplementary Table 3.** Genotype distribution and Hardy–Weinberg equilibrium in the overall sample (*n* = 396).

| SNP | Genotype (*n*, %) | Major allele | Minor allele | Call rate (%) | HWE 𝑝-value |
| --- | --- | --- | --- | --- | --- |
| APOA1 rs670 | TT: 34 ( 8.6%) | C | T | 100 | 0.574 |
|  | CT: 172 (43.4%) |  |  |  |  |
|  | CC: 190 (48.0%) |  |  |  |  |
|  |  |  |  |  |  |
| LIPC rs1800588 | CC: 90 (22.7%) | T | C | 100 | 0.733 |
|  | CT: 194 (49.0%) |  |  |  |  |
|  | TT: 112 (28.3%) |  |  |  |  |
|  |  |  |  |  |  |
| CETP rs708272 | AA: 89 (22.5%) | G | A | 100 | 0.053 |
|  | AG: 158 (39.9%) |  |  |  |  |
|  | GG: 149 (37.6%) |  |  |  |  |

Genotype frequencies are presented as numbers (percentages). Major and minor alleles were defined based on overall sample distribution. The Hardy–Weinberg equilibrium (HWE) was tested in the overall sample using the *χ*^2^ goodness-of-fit test.

**Supplementary Table 4.** Partial correlation between AIP and TyG and component-level regression models for triglycerides and HDL-C.

| Predictor | TG (z-score) | | | |  | HDL-C (z-score) | | | |  | AIP-TyG association | |
| --- | --- | --- | --- | --- | --- | --- | --- | --- | --- | --- | --- | --- |
|  | *β* | SE | 95% CI | 𝑝-value |  | *β* | SE | 95% CI | 𝑝-value |  | *β* | 𝑝-value |
| Intercept | −0.4598 | 0.2425 | [−0.9366, 0.0169] | 0.059 |  | −0.3236 | 0.2471 | [−0.8094, 0.1621] | 0.191 |  |  |  |
| WHtR | **0.2163** | 0.0578 | [ 0.1026, 0.3300] | **< 0.001** |  | **−0.3941** | 0.0589 | [−0.5100, −0.2783] | **< 0.001** |  |  |  |
| wGRS | **−0.1410** | 0.0488 | [−0.2369, −0.0451] | **0.004** |  | −0.0676 | 0.0497 | [−0.1653, 0.0301] | 0.174 |  |  |  |
| WHtR × wGRS interaction | **0.1310** | 0.0504 | [ 0.0319, 0.2302] | **0.010** |  | −0.0783 | 0.0514 | [−0.1794, 0.0227] | 0.128 |  |  |  |
| Age (years) | 0.0116 | 0.0039 | [ 0.0039, 0.0192] | 0.003 |  | 0.0042 | 0.0040 | [−0.0036, 0.0120] | 0.291 |  |  |  |
| Sex (male = 1) | −0.2227 | 0.1118 | [−0.4426, −0.0029] | 0.047 |  | −0.1569 | 0.1139 | [−0.3810, 0.0671] | 0.169 |  |  |  |
| Fasting Status | 0.0794 | 0.2009 | [−0.3156, 0.4744] | 0.693 |  | 0.2440 | 0.2047 | [−0.1585, 0.6464] | 0.234 |  |  |  |
| Lipid-lowering medication | 0.0114 | 0.1475 | [−0.2786, 0.3015] | 0.938 |  | −0.1693 | 0.1503 | [−0.4649, 0.1263] | 0.261 |  |  |  |
| Glucose-lowering medication | 0.0746 | 0.1790 | [−0.2773, 0.4264] | 0.677 |  | −0.2276 | 0.1824 | [−0.5862, 0.1309] | 0.213 |  |  |  |
| Antihypertensive medication | 0.1764 | 0.1830 | [−0.1834, 0.5362] | 0.336 |  | 0.2085 | 0.1865 | [−0.1581, 0.5752] | 0.264 |  |  |  |
| Alcohol consumption (yes = 1) | 0.1625 | 0.1064 | [−0.0466, 0.3716] | 0.127 |  | −0.1064 | 0.1084 | [−0.3195, 0.1067] | 0.327 |  |  |  |
| Spearman ρ (AIP, TyG) |  |  |  |  |  |  |  |  |  |  | **0.8794** | **< 0.001** |
| r (AIP, TyG \| TG) |  |  |  |  |  |  |  |  |  |  | **−0.1178** | **0.019** |

Spearman’s correlation and partial correlation controlling for triglycerides are presented in the first row. Regression models included WHtR_z_, wGRS_z_, their interaction term, and adjustment for age, sex, fasting status, lipid-lowering medication, glucose-lowering medication, antihypertensive medication and self-reported alcohol consumption (no/yes). WHtR and wGRS were analyzed as z-standardized variables.

**Supplementary Table 5.** Multivariable linear regression models evaluating the interaction of WHtR × wGRS with AIP.

| Predictor | Model 1 (Original scale) | | | |  | Model 2 (z-standardized) | | | |  | VIF |
| --- | --- | --- | --- | --- | --- | --- | --- | --- | --- | --- | --- |
|  | *β* | SE | 95% CI | 𝑝-value |  | *β* | SE | 95% CI | 𝑝-value |  |  |
| Intercept | 0.2873 | 0.1275 | [ 0.0367, 0.5379] | 0.025 |  | 0.3871 | 0.0613 | [ 0.2665, 0.5077] | < 0.001 |  |  |
| WHtR | 0.2573 | 0.1957 | [−0.1275, 0.6421] | 0.189 |  | **0.1031** | 0.0146 | [ 0.0744, 0.1319] | **< 0.001** |  | 1.59 |
| wGRS | −3.5268 | 0.9017 | [−5.2996, −1.7539] | < 0.001 |  | **−0.0293** | 0.0123 | [−0.0536, −0.0051] | **0.018** |  | 1.13 |
| WHtR × wGRS interaction | 5.4291 | 1.5408 | [ 2.3996, 8.4586] | < 0.001 |  | **0.0449** | 0.0128 | [ 0.0199, 0.0700] | **< 0.001** |  | 1.02 |
| Age (years) | 0.0025 | 0.0010 | [ 0.0006, 0.0044] | 0.012 |  | 0.0025 | 0.0010 | [ 0.0006, 0.0044] | 0.012 |  | 1.41 |
| Sex (male = 1) | −0.0316 | 0.0283 | [−0.0872, 0.0240] | 0.264 |  | −0.0316 | 0.0283 | [−0.0872, 0.0240] | 0.264 |  | 1.05 |
| Fasting Status | −0.0116 | 0.0508 | [−0.1115, 0.0883] | 0.819 |  | −0.0116 | 0.0508 | [−0.1115, 0.0883] | 0.819 |  | 1.01 |
| Lipid-lowering medication | −0.0073 | 0.0373 | [−0.0807, 0.0661] | 0.845 |  | −0.0073 | 0.0373 | [−0.0807, 0.0661] | 0.845 |  | 1.03 |
| Glucose-lowering medication | 0.0572 | 0.0453 | [−0.0318, 0.1461] | 0.207 |  | 0.0572 | 0.0453 | [−0.0318, 0.1461] | 0.207 |  | 1.07 |
| Antihypertensive medication | −0.0093 | 0.0463 | [−0.1003, 0.0816] | 0.840 |  | −0.0093 | 0.0463 | [−0.1003, 0.0816] | 0.840 |  | 1.05 |
| Alcohol consumption (yes = 1) | 0.0568 | 0.0269 | [ 0.0039, 0.1097] | 0.035 |  | 0.0568 | 0.0269 | [ 0.0039, 0.1097] | 0.035 |  | 1.03 |

Model 1 presents the unstandardized coefficients. Model 2 was estimated using the z-standardized WHtR and wGRS; the interaction term corresponded to WHtR_z_ × wGRS_z_. The models were adjusted for age, sex, fasting status, lipid-lowering medication, glucose-lowering medication, antihypertensive medication, and self-reported alcohol consumption (no/yes). Variance inflation factors (VIF) were calculated using model 2. Two-sided 𝑝-values are reported.

**Supplementary Table 6.** Multivariable linear regression model of WHtR × wGRS interaction on AIP in women.

| Predictor | *β* | SE | 95% CI | 𝑝-value |
| --- | --- | --- | --- | --- |
| Intercept | 0.3898 | 0.0790 | [ 0.2343, 0.5454] | < 0.001 |
| WHtR | **0.1083** | 0.0172 | [ 0.0744, 0.1421] | **< 0.001** |
| wGRS | −0.0177 | 0.0147 | [−0.0465, 0.0111] | 0.228 |
| WHtR × wGRS interaction | **0.0365** | 0.0153 | [ 0.0065, 0.0665] | **0.017** |
| Age (years) | 0.0035 | 0.0016 | [ 0.0004, 0.0065] | 0.026 |
| Fasting Status | −0.0243 | 0.0565 | [−0.1354, 0.0869] | 0.668 |
| Lipid-lowering medication | 0.0170 | 0.0428 | [−0.0672, 0.1012] | 0.692 |
| Glucose-lowering medication | 0.0548 | 0.0586 | [−0.0605, 0.1700] | 0.351 |
| Antihypertensive medication | 0.0410 | 0.0538 | [−0.0648, 0.1469] | 0.446 |
| Menopausal status | −0.0985 | 0.0466 | [−0.1903, −0.0067] | 0.035 |
| Hormonal therapy | −0.0151 | 0.0858 | [−0.1840, 0.1539] | 0.861 |
| Contraceptive use | −0.0492 | 0.0340 | [−0.1161, 0.0177] | 0.149 |
| Alcohol consumption (yes =1) | 0.0621 | 0.0325 | [−0.0019, 0.1261] | 0.057 |

The predictors were z-standardized. The interaction term corresponds to the product of WHtR and wGRS. The model was adjusted for age, fasting status, lipid-lowering medication, glucose-lowering medications, antihypertensive medications, menopausal status, hormonal therapy, contraceptive use, and self-reported alcohol consumption (no/yes). Sex was not included because the analysis was restricted to women. Two-sided 𝑝-values are reported.

**Supplementary Table 7.** Multivariable linear regression model of WHtR × wGRS interaction on TyG.

| Predictor | *β* | SE | 95% CI | 𝑝-value |
| --- | --- | --- | --- | --- |
| Intercept | 8.3465 | 0.1530 | [ 8.0457, 8.6473] | < 0.001 |
| WHtR | **0.1876** | 0.0365 | [ 0.1159, 0.2593] | **< 0.001** |
| wGRS | **−0.0882** | 0.0308 | [−0.1487, −0.0277] | **0.004** |
| WHtR × wGRS interaction | **0.1026** | 0.0318 | [ 0.0401, 0.1652] | **0.001** |
| Age (years) | 0.0093 | 0.0025 | [ 0.0044, 0.0141] | < 0.001 |
| Sex (male = 1) | −0.1350 | 0.0706 | [−0.2737, 0.0038] | 0.057 |
| Fasting Status | 0.0115 | 0.1268 | [−0.2377, 0.2608] | 0.927 |
| Lipid-lowering medication | −0.0957 | 0.0931 | [−0.2787, 0.0873] | 0.305 |
| Glucose-lowering medication | 0.0660 | 0.1129 | [−0.1561, 0.2880] | 0.559 |
| Antihypertensive medication | 0.1706 | 0.1155 | [−0.0565, 0.3976] | 0.140 |
| Alcohol consumption (yes = 1) | 0.1183 | 0.0671 | [−0.0136, 0.2503] | 0.079 |

The predictors were z-standardized. The interaction term corresponds to the product of WHtR and wGRS. The model was adjusted for age, sex, fasting status, lipid-lowering medications, glucose-lowering medications, antihypertensive medications, and self-reported alcohol consumption (no/yes). Two-sided 𝑝-values are reported.

**Supplementary Table 8.** Sex-stratified quantile regression models of the AIP at 0.75 and 0.90 quantiles.

| Sex | Quantile | N |  | WHtR | |  | wGRS | |  | WHtR × wGRS | |
| --- | --- | --- | --- | --- | --- | --- | --- | --- | --- | --- | --- |
|  |  |  |  | *β* | 𝑝-value |  | *β* | 𝑝-value |  | *β* | 𝑝-value |
| Men | 0.75 | 90 |  | 0.3275 | 0.002 |  | −0.1583 | 0.204 |  | **0.3122** | **0.002** |
|  | 0.90 | 90 |  | 0.0654 | 0.560 |  | −0.1782 | 0.103 |  | 0.1478 | 0.070 |
|  |  |  |  |  |  |  |  |  |  |  |  |
| Women | 0.75 | 306 |  | 0.5118 | < 0.001 |  | 0.0352 | 0.683 |  | **0.1656** | **0.028** |
|  | 0.90 | 306 |  | 0.4373 | < 0.001 |  | −0.0548 | 0.527 |  | 0.0372 | 0.638 |

Quantile regression models were estimated separately by sex at the 0.75 and 0.90 quantiles of AIP (z-score). WHtR and wGRS were z-standardized, and the interaction term corresponded to their product. Models additionally included self-reported alcohol consumption (no/yes) as a covariate. Two-sided 𝑝-values are reported.

**Supplementary Table 9.** Minimum detectable effects for the sex-stratified WHtR × wGRS interaction models.

| Sex | N | Residual df | *β* | SE | 95% CI | 𝑝-value | MDE at 80% power |
| --- | --- | --- | --- | --- | --- | --- | --- |
| Men | 90 | 80 | 0.2649 | 0.0822 | [ 0.1013, 0.4284] | 0.002 | 0.2331 |
| Women | 306 | 296 | 0.0870 | 0.0573 | [−0.0257, 0.1998] | 0.130 | 0.1610 |

Minimum detectable effects (MDEs) were calculated for 80% power at a two-sided α of 0.05. β and MDE are expressed in AIP z-score units for the WHtR × wGRS interaction. Models were adjusted for age, fasting status, lipid-lowering, glucose-lowering and antihypertensive medication, and self-reported alcohol consumption (no/yes). Sex-stratified analyses were considered exploratory. AIP – atherogenic index of plasma; WHtR – waist-to-height ratio; wGRS – weighted genetic risk score; df – degrees of freedom; SE – standard error; CI – confidence interval; MDE – minimum detectable effect.

**Supplementary Table 10.** Sex-stratified adjusted AIP estimates across standardized WHtR and wGRS values.

| Sex | WHtR (z-score) | wGRS (z-score) | AIP (z-score) | SE | 95% CI |
| --- | --- | --- | --- | --- | --- |
| Men | −1 SD | −1 SD | 0.2374 | 0.1896 | [−0.1398, 0.6147] |
|  |  | Mean | −0.2164 | 0.1267 | [−0.4685, 0.0357] |
|  |  | +1 SD | −0.6702 | 0.1552 | [−0.9790, −0.3615] |
|  |  |  |  |  |  |
|  | Mean | −1 SD | 0.2586 | 0.1227 | [ 0.0143, 0.5028] |
|  |  | Mean | 0.0696 | 0.0878 | [−0.1052, 0.2443] |
|  |  | +1 SD | −0.1194 | 0.1232 | [−0.3645, 0.1257] |
|  |  |  |  |  |  |
|  | +1 SD | −1 SD | 0.2797 | 0.1469 | [−0.0126, 0.5719] |
|  |  | Mean | 0.3555 | 0.1305 | [ 0.0957, 0.6153] |
|  |  | +1 SD | 0.4314 | 0.2031 | [ 0.0273, 0.8355] |
|  |  |  |  |  |  |
| Women | −1 SD | −1 SD | −0.1951 | 0.1299 | [−0.4507, 0.0605] |
|  |  | Mean | −0.3556 | 0.0802 | [−0.5133, −0.1978] |
|  |  | +1 SD | −0.5160 | 0.0952 | [−0.7033, −0.3287] |
|  |  |  |  |  |  |
|  | Mean | −1 SD | 0.1026 | 0.0808 | [−0.0565, 0.2617] |
|  |  | Mean | 0.0292 | 0.0569 | [−0.0828, 0.1412] |
|  |  | +1 SD | −0.0443 | 0.0811 | [−0.2039, 0.1153] |
|  |  |  |  |  |  |
|  | +1 SD | −1 SD | 0.4004 | 0.0922 | [ 0.2190, 0.5817] |
|  |  | Mean | 0.4139 | 0.0822 | [ 0.2521, 0.5757] |
|  |  | +1 SD | 0.4274 | 0.1355 | [ 0.1609, 0.6940] |

Values are adjusted predicted z-scores with standard errors and 95% confidence intervals. WHtR and wGRS were evaluated at −1 SD, the mean, and +1 SD. Models were adjusted for age, fasting status, lipid-lowering, glucose-lowering and antihypertensive medication, and self-reported alcohol consumption (no/yes). AIP – atherogenic index of plasma; WHtR – waist-to-height ratio; wGRS – weighted genetic risk score; SD – standard deviation; SE – standard error; CI – confidence interval.
